# Qualitative Behavioural Assessment as a welfare indicator for farmed Atlantic salmon (*Salmo salar)* in response to a stressful challenge

**DOI:** 10.3389/fvets.2023.1260090

**Published:** 2023-09-28

**Authors:** Timothy Robert Wiese, Sonia Rey Planellas, Monica Betancor, Marie Haskell, Susan Jarvis, Andrew Davie, Francoise Wemelsfelder, James F. Turnbull

**Affiliations:** ^1^Institute of Aquaculture, University of Stirling, Stirling, United Kingdom; ^2^Scotland’s Rural College SRUC, Edinburgh, United Kingdom; ^3^Global Academy of Agriculture and Food Systems, University of Edinburgh, Midlothian, United Kingdom; ^4^Aquascot Ltd., Alness, United Kingdom

**Keywords:** emotional state, aquaculture, positive welfare, behavioural analysis, qualitative behaviour assessment

## Abstract

Animal welfare assessments have struggled to investigate the emotional states of animals while focusing solely on available empirical evidence. Qualitative Behavioural Assessment (QBA) may provide insights into an animal’s subjective experiences without compromising scientific rigor. Rather than assessing explicit, physical behaviours (i.e., what animals are doing, such as swimming or feeding), QBA describes and quantifies the overall expressive manner in which animals execute those behaviours (i.e., how relaxed or agitated they appear). While QBA has been successfully applied to scientific welfare assessments in a variety of species, its application within aquaculture remains largely unexplored. This study aimed to assess QBA’s effectiveness in capturing changes in the emotional behaviour of Atlantic salmon following exposure to a stressful challenge. Nine tanks of juvenile Atlantic salmon were video-recorded every morning for 15 min over a 7-day period, in the middle of which a stressful challenge (intrusive sampling) was conducted on the salmon. The resultant 1-min, 63 video clips were then semi-randomised to avoid predictability and treatment bias for QBA scorers. Twelve salmon-industry professionals generated a list of 16 qualitative descriptors (e.g., relaxed, agitated, stressed) after viewing unrelated video-recordings depicting varying expressive characteristics of salmon in different contexts. A different group of 5 observers, with varied experience of salmon farming, subsequently scored the 16 descriptors for each clip using a Visual Analogue Scale (VAS). Principal Components Analysis (correlation matrix, no rotation) was used to identify perceived patterns of expressive characteristics across the video-clips, which revealed 4 dimensions explaining 74.5% of the variation between clips. PC1, ranging from ‘relaxed/content/positive active’ to ‘unsettled/stressed/spooked/skittish’ explained the highest percentage of variation (37%). QBA scores for video-clips on PC1, PC2, and PC4 achieved good inter- and intra-observer reliability. Linear Mixed Effects Models, controlled for observer variation in PC1 scores, showed a significant difference between PC1 scores before and after sampling (*p* = 0.03), with salmon being perceived as more stressed afterwards. PC1 scores also correlated positively with darting behaviours (*r* = 0.42, *p* < 0.001). These results are the first to report QBA’s sensitivity to changes in expressive characteristics of salmon following a putatively stressful challenge, demonstrating QBA’s potential as a welfare indicator within aquaculture.

## 1. Introduction

Animal welfare science has faced the challenge of addressing all aspects of welfare without compromising objectivity and the need for empirical evidence. Physical health has long been recognised as an essential component of animal welfare (1–3). However, a widely held perspective now is that animal welfare is ultimately a state that is perceived by the animal itself, and we should therefore also include concerns for the animal’s mental well-being (4–6). There is thus a growing demand that welfare assessments, including those for fish, adopt a more holistic approach that places additional focus on monitoring the animal’s positive experiences (2, 6–11). Welfare appraisals that adopt this integrated approach, however, inevitably enter the murky waters that are an animal’s subjective experiences (12, 13). Despite decades of research trying to resolve this issue, the only progress thus far has been reaching a consensus that there is no single “measure” that can adequately cover what welfare entails (13–16). This dilemma has resulted in the mental well-being of fish often being overlooked in welfare assessments (17).

In 2018, Atlantic salmon accounted for 4.5% of global aquaculture production by tonnage (18). In 2021, production of Scottish Atlantic salmon reached an all-time high of 205,393 tonnes, with more than 50 million smolts transferred to sea in the same year (19). Total tonnage of Scottish farmed salmon, relative to the number of employees on-site, has increased 11-fold within seawater and 6-fold within freshwater between 1985 and 2016 (20). This increase in the numbers of fish relative to farm staff, unavoidably reduces the time available for monitoring the salmon. There is also mounting scientific evidence supporting the sentience of fish (21–24). A UK National survey, involving 1963 members of the public, found that 77% agreed or strongly agreed that fish can feel pain, and 80% agreed that this should therefore be of concern (25). Considering the scale of this industry, there is a clear ethical and economic incentive to develop welfare indicators that are not only practical, but attempt to include aspects of mental well-being (both positive and negative) in their assessment.

To achieve such an assessment, a framework was proposed in which welfare assessments are viewed in the context of a simple question: “Is the animal healthy, and does it have what it wants?” (13). Answering the second, difficult part of this question (i.e., delving into an animal’s subjective experiences) may require accepting two arguments. Firstly, that consciousness still presents an impasse for scientific study (3). Secondly, given that animals cannot express their desires/needs in human language, behavioural analysis may provide some of the best insights into what they “want” (12). Behaviours exhibited by an animal are, in essence, the final product of all its own decision-making processes (1, 26). They are the “final common path,” as described by Sherrington (27), or, in Charles Darwin’s words, the “ultimate phenotype” and “expression of the emotions” (27). Behavioural analysis provides a number of additional advantages over physiological/morphological measures in welfare assessments. Such analyses are frequently non-intrusive (the animal is unaware it is being assessed), and are often quick to observe (1, 28, 29). Behaviour is also gaining recognition as a general, pre-clinical ‘early warning system’ for issues that may be emerging within the stock (1, 28, 30–32).

Observant farmers are capable of detecting changes in the demeanour and behaviour of their animals (33). Such knowledge, typically gained through years of experience, enables farmers to detect subtle shifts in how animals express themselves when issues arise, even though the exact nature of the problem may remain unclear (34). Qualitative Behavioural Assessment (QBA) is a behavioural assessment tool that benefits from this approach, with its reproducibility and validity demonstrated in previous research (35). QBA is an integrative assessment of the “whole-animal,” where observations are made on the animal’s dynamic body language (including their appearance, behaviour, and interaction with others and the surrounding environment) as an indicator of its welfare state (36–39). Different aspects of this body language are summarised through a number of ‘descriptors’ (or terms) such as: relaxed, inquisitive, agitated, or stressed (17, 36, 40). Such terms focus not so much on what an animal does (e.g., feeding), as on how it does this; the expressive characteristics shown in the way it moves (40). Descriptions of such characteristics typically have an emotional connotation, indicating how the animal is experiencing the situation it is in, and QBA is therefore hypothesized to be able to contribute valuable information on an animal’s emotional state to studies of animal welfare. Interest in the study of fish emotion has grown in recent years. A variety of reviews conclude that fishes are neuro-physiologically and behaviourally similar enough to mammals to warrant assuming a comparable emotional range in fishes, both in terms of negative and positive emotions (6, 41, 42). Indeed, fish are found to be so intelligent that they are frequently used as models for the study of cognition in mammals (43). There is thus a need for developing and testing methods able to address emotional behaviour in fish, and QBA has been recognised as one such potentially promising method (11).

Previous studies for various livestock species have validated the use of QBA against other welfare indicators, and demonstrated high degrees of inter-observer reliability between observers (33, 44). Additionally, QBA allows for simple, time-efficient, and non-intrusive assessments of an animal’s well-being (35, 39). QBA is also the only measure currently included in the EU Welfare Quality® welfare assessment protocols to assess positive emotional states in cattle, pigs, and poultry (45, 46). To date, however, the only QBA study to be applied to fish examined solely the inter/intra-observer reliability and QBA’s association with ethograms of salmon behaviour, without the inclusion of any treatments (17). No studies have yet examined fish exposed to stressors, or compared QBA scores in this context to other welfare indicators. Comparing QBA scores against other welfare indicators for salmon may help to further explore what potential role QBA may have as a welfare assessment tool. Darting represents a behavioural response previously recorded in fear-conditioning studies of fish, and is commonly associated with predator avoidance (47–50). It is considered a stress response which, when increasing in frequency/intensity, may indicate impaired welfare (47, 48, 51). Feed intake is also generally considered a reliable indicator within health and welfare assessments of farmed fish (52). A loss in appetite is potentially a sign of impaired welfare (28, 53). The main aim of this study was therefore to examine QBA’s ability to detect differences in the expressive characteristics of Atlantic salmon after exposure to a stressful challenge (i.e., an intrusive sampling event). In addition, this study also aimed to compare these QBA scores against other welfare indicators for salmon; their daily feed intake (as a proxy for appetite) and darting behaviours (i.e., sudden, rapid movements of the salmon).

## 2. Materials and methods

### 2.1. Ethical review

Ethical approval for the recording of salmon and QBA work was obtained from the University of Stirling’s Animal Welfare & Ethical Review Body (Approval reference no. 2022-6783-5196).

### 2.2. Experimental set-up

#### 2.2.1. Animals

The juvenile Atlantic salmon used in this study were transferred on November 16^th^, 2021, from the Niall Bromage Freshwater Research Unit (NBFRU), Denny, to the Marine Environmental Research Laboratory (MERL) in Campbeltown, Argyll and Bute, Scotland. The salmon were around 14 months of age, and weighed on average 285–360 grams. There were ~ 80 smolts in each tank at the start of the recording, with an average stocking density of ~34 kg/m^3^.

#### 2.2.2. Husbandry

The salmon were housed in a total of 9 identical flow-through tanks (1.4 m diameter, 750 L volume). Seawater was filtered through a Lacron sand filter (4×100 micron bag filters) before flowing into the tanks to minimise turbidity. Feed was formulated to satisfy the nutrient requirements for Atlantic salmon (54) and contained 46% protein and 24% fat. Automatic belt feeders provided formulated diet salmon pelleted dry feed to all tanks every 20 min between 05:00–09:00 and 16:30–23:30. Dirty water and uneaten feed were flushed out of the tanks through standpipes daily, between 09:00 and 09:15. Any mortalities found during this period were immediately removed. Lights were turned on at exactly 10:30 am each morning.

#### 2.2.3. Treatments (including stressful challenge)

Video clips for this study were recorded around a stressful challenge, conducted on February 18th, 2022. This stressful challenge involved a sampling event which was carried out for another study on these salmon. This required capturing, anaesthetising, and handling each of the salmon out of water for measuring their weight, length, and condition factor. While feed withdrawal was also required 24 h before sampling could be carried out, the recording schedule was designed on the assumption that the main disturbances (i.e., stressful challenge) to the salmon would occur largely as a result of this sampling event. For the purposes of the study that involved the sampling event, a subset of the salmon that were sampled were then euthanised. Fish were euthanised with anaesthetic overdose of Tricaine Methanesulfonate (MS-222) and pithing (Schedule 1) in order to obtain their hepatosomatic index. Following the sampling event, there were approximately 50 salmon left in each tank, with an average stocking density of ~21 kg/m^3^.

#### 2.2.4. Camera and tanks set-up

Cameras were installed in the tanks to record video clips for the QBA and behavioural assessments. To do this, every morning at 9 am, GoPro Hero9 Black© cameras were installed at 1 m depth using a fixed metal pole, which was positioned flush against the inside of each tank to ensure the same angle and field of view (FOV) for recordings. This was carried out 90 min before lights went on to allow time for salmon to habituate to the cameras. These cameras were also installed each morning for 2 days before recording commenced to allow the salmon to further habituate to these novel objects. To minimise any additional disturbances, cameras were turned on before being submerged with recording controlled remotely through the GoPro Quik© mobile application. Connectivity from mobile phone to each underwater camera was achieved through the use of coaxial cables taped to each device. Coaxial cables conduct electrical signals (including Wi-Fi) through an insulated shield, extending network connections to a submerged device (e.g., camera). Recordings for each tank were taken on a strict daily schedule, after lights went on, to ensure consistency. A minimum of 15 min were recorded for each tank once lights went on. All personnel on-site strictly avoided carrying out any procedures around the tanks during filming.

#### 2.2.5. Recording schedule

**Figure 1:**
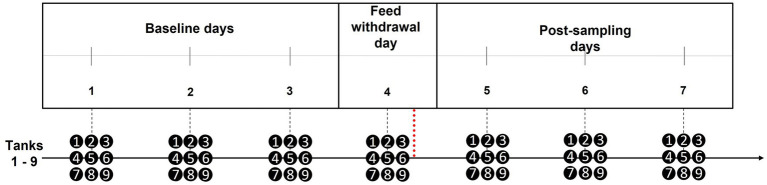
Recording schedule and timeline for experiment. Black dots represent each time a tank was recorded for the day, and the dashed red line (after day 4) illustrates when the stressful challenge (sampling event) occurred.

A 7-day period of video recording was scheduled to gather footage for all behavioural analysis (i.e., QBA and darting behaviours), with the stressful challenge (i.e., sampling) conducted during the middle of this period. Sampling was carried out on all 9 tanks of salmon on February 18^th^, 2022. To obtain a ‘baseline’ and account for any potential day to day variation in behaviour, 3 consecutive days were recorded before the stressful challenge occurred. A further 3 consecutive ‘post-sampling’ days were required for recording the salmon’s recovery from this stressful challenge. Figure 1 provides a summary of the recording schedule. The ability for QBA to reflect any impacts on salmon behavioural expressions, as a result of these disturbances, could then be assessed from these recordings (Section 2.3.1.1 outlines how the video clips were prepared for QBA).

### 2.3. Qualitative Behavioural Assessment

The QBA process consisted of two main stages. Stage 1 involved 12 observers in the generation of the QBA terms for describing the salmon’s expressive characteristics and stage 2 involved 5 different observers scoring the QBA terms for each of the video clips.

#### 2.3.1. Stage 1 – term generation

**Figure 2:**
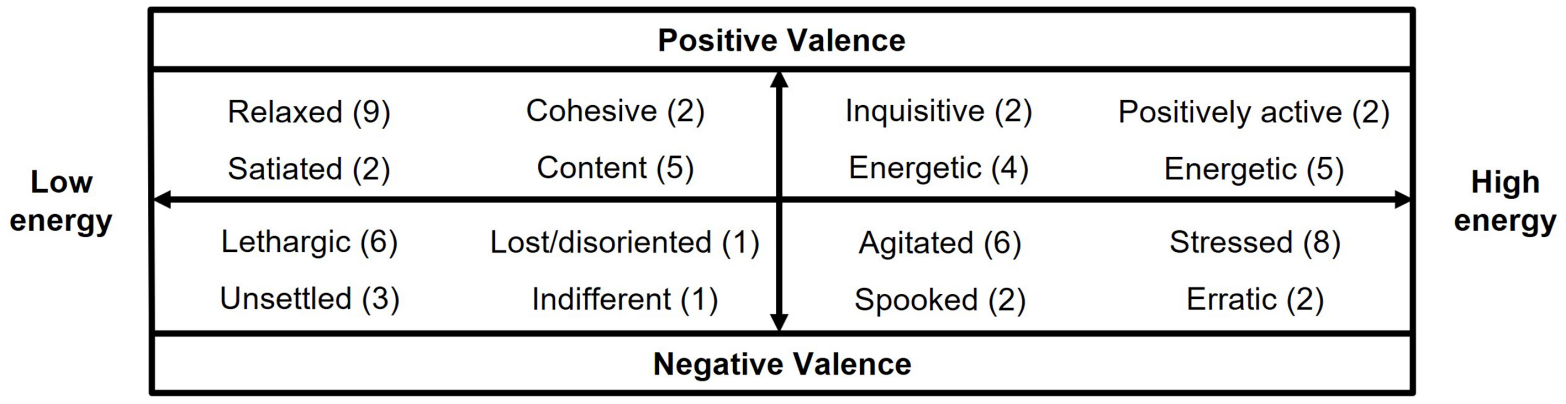
Final list of QBA terms generated from stage 1. Valence (positive/negative) and energy (high/low) were used to help describe and discuss terms across the 4 quadrants. Numbers in brackets indicate the total number of participants who brought each term to the initial meeting.

Twelve professionals employed in the Scottish salmon farming industry were recruited for the term generation stage, which involved two separate meetings. All participants had at least 1 year of experience working directly with farmed salmon, with a number of participants in senior/management roles. During term generation, various video clips were used which were taken from different farm sites under different contexts (e.g., during the middle of the day or during feeding, after treatments/transportation etc.). In this study, we define ‘expressive characteristics’ as the extent to which qualitative characteristics of salmon behaviour (e.g., relaxed, purposeful, lethargic, agitated) are expressed. The video clips were selected to represent all 4 aspects, or ‘quadrants’, of behavioural expression (high to low energy, positive to negative valence) as outlined by Mendl et al. (26) (see Figure 2).

Before terms were generated by participants, the theory and practice of QBA was explained to them and they were provided with guidance on how to generate appropriate terms. To avoid bias, examples of terms from terrestrial farming systems were used. After the first meeting, participants were asked to individually watch the video clips in advance of the second meeting and generate their own personal list of terms. During the second meeting the participants discussed these terms, including how they should be divided between the 4 quadrants of behavioural expression (high to low energy, positive to negative valence). Participants were then asked to select a maximum of 20 terms which were balanced across the 4 quadrants, and best described the range of salmon behavioural expression. By the end of the meeting, the group had agreed on 16 terms. These included the terms “diving deep” and “flighty,” which were excluded by the experimenters from the final list used in the second stage. QBA requires terms that convey some aspect of emotional behaviour and the term “deep diving” did not. Other terms (e.g., spooked, erratic, unsettled, agitated) already covered aspects of the term “flighty.” The final QBA term list therefore had 16 terms, with 4 in each quadrant of behavioural expression (Figure 2). These terms were then used in the QBA scoring stage.

##### 2.3.1.1. Video preparation before stage 2

For use in the QBA scoring stage, shorter video clips were extracted from each of the 63, 15-min videos. These clips were the first full minute that the salmon remained clearly in view, starting from 30 s after the lights were turned on. This excluded the initial “noise” from the salmon’s startle responses to the lights. Video clips were first randomised with respect to their chronological order and their occurrence before or after the sampling treatment. To facilitate observer concentration and motivation, they were then arranged so that clips showing contrasting expressive characteristics (e.g., primarily high energy, negative valence vs. low energy, positive valence) were distributed evenly throughout the scoring sessions. Unknown to observers, 4 of the original 63 video clips were duplicated to allow for an assessment of intra-observer reliability (the degree to which participants showed agreement within their own scoring sessions). This resulted in a total of 67 video clips being scored by each observer.

#### 2.3.2. Stage 2 – QBA training and scoring session

Scoring sessions for the QBA were carried out with a new group of 5 observers. These 5 observers consisted of 2 Post-doctoral fish welfare researchers from the University of Stirling, and 3 industry professionals all with higher degree education and between 3 and 20 years of aquaculture industry experience. These observers consequently had a varied level of experience in working with and observing salmon. All but 1 observer had hands-on experience in salmon husbandry in a commercial setting.

Observers were given online training in QBA. A brief introduction was given on the principles of QBA and the general purpose of this study (i.e., exploring the use of QBA within fish). Observers were kept blind to treatment (i.e., the stressful sampling challenge), and were instead only informed about the general context behind the video clips (location of filming, number of tanks and days involved in the recording). It was explained to observers that qualitative descriptors overlap and complement each other in characterising expressive patterns and are not mutually exclusive in the way ethogram categories are. To capture subtle differences when scoring it is important to consider the meaning of each individual descriptor in its own right. Associations between different terms are complex; fish could for example appear stressed and spooked but not too agitated, or a bit unsettled and agitated but not too stressed. To support such use of the descriptors it is important that everyone’s understanding of the terms is aligned as much as possible. To this end, an open discussion of the meaning of terms was conducted, aided by a sheet with brief characterisations of each term created by the experimenters (Table 1). The meaning of each term was discussed and adjusted where required, and observers were invited to raise any questions about terms which required clarification. General instructions were given on how to assess whole animal expressivity and how to use the Visual Analogue Scales (VAS) to score the prevalence of each term within a video clip. A VAS is a measurement instrument that allows for the scoring of characteristics (such as those of behavioural expressions) that are believed to range across a continuum of values (55). Observers were reminded that terms must be scored independently from each other, so that in situations where there were contrasting expressive characteristics among different salmon (e.g., some appearing agitated and others relaxed), those contrasting terms could both receive high scores for the same clip.

**Table 1:** QBA term list with term characterisations.

Term	Term characterisation
Relaxed	Salmon are moving at ease, free from tension or agitation. Does not just apply to resting - animals can be relaxed also while active.
Agitated	Salmon are restless, excessively moving around, over-responding to unexpected stimuli.
Inquisitive	Salmon show an interest/curiosity towards their surroundings - explorative, investigating features (novel or familiar) of their environment.
Unsettled	Salmon are uncertain, ill at ease, twitchy, vigilant.
Cohesive	Salmon are moving together in synchrony/unison; the shoal appears to behave as one organism.
Spooked/skittish	Salmon are easily scared en-masse (even by small disturbances), abruptly changing behaviour/direction of travel, and avoiding rather than investigating.
Positive active	Salmon are absorbed in activity in a relaxed way, interacting in a positive manner with their environment.
Indifferent	Salmon are unfocused, moving around without much engagement, lacklustre. Not dull or lethargic.
Purposeful	Salmon are self-motivated, focused, determined. Carrying out their actions without hesitation.
Erratic	Salmon movements are un-coordinated, randomly (over)reacting, disorganised. Erratic is more a more vigorous expression than being unsettled.
Energetic	Salmon move in a vigorous, lively way; appearing bright & animated.
Lost/disoriented	Salmon do not know what to do, appearing aimless, confused, worried, and searching on their own.
Satiated	Salmon appear to be satisfied in their physical needs.
Lethargic	Salmon are dull, morose, unresponsive, slow-moving and without any vigour. Looking unwell.
Stressed	Salmon are troubled, tense, not behaving normally. Something is not right.
Content	Salmon are healthy, calm, satisfied, looking well. At ease but could lack positive engagement/purpose.

Terms are listed in the order in which they were presented for scoring to observers.

All QBA scoring was carried out on scoring sheets developed on SurveyMonkey®. For each term, a horizontal line with a 100-step scale was presented as a VAS, along which a single mark could be made. The distance from the left end of the scale would correspond to the participant’s assessment of the intensity for each term observed. The left end of the scale represented complete absence of an expressive characteristic described by a term, whereas the right end represented the maximum expression for the term (e.g., the salmon could not be more erratic). While scoring, observers would not be aware of any quantitative values associated with the VASs. They were encouraged to use the entire scale when judging the intensity of each expressive characteristic. Video clips were labelled according to their order in the scoring sheets and transferred electronically to the group. Due to the large number of clips, observers were instructed to avoid scoring them all in a single session, but also to carry out their scoring sessions with minimal delay between each other (i.e., within the same week) to minimise potential variation introduced by scoring on different days.

### 2.4. Additional welfare measures – feed intake and darting events

#### 2.4.1. Feed intake

Feed input and feed waste were recorded for each tank daily alongside the 7 days of QBA recordings. The experimental feeds were given by auto-feeders (Arvo-tec TD2000) twice a day from 05:00–09:00 and 16:30–23:30 with uneaten feed collected to measure daily feed intake and calculate apparent feed intake through standpipes daily, between 09:00 and 09:15. Recovered feeds were placed in a 300 μm mesh sieve and rinsed in fresh water in order to remove salt and faeces. The leaching of the feeds was calculated through a nutrient dissolution factor. Briefly, 10 g of each feed was incubated in system water in duplicates for 6 h before drying (24 h, 110°C) and weighing. Feed waste daily collected was weighed wet and converted to dry weight using the nutrient dissolution factor.

#### 2.4.2. Darting behaviour

For the purpose of this study, darting behaviours were defined as a “rapid, burst of movement clearly distinct from the salmons’ regular swimming behaviours; this includes sudden changes in direction, acceleration, and/or positioning of the salmon in the tank.” A scan sampling method (56) was created to record ‘darting events’ in the same 63, 1-min video clips used for the QBA. Since any of these darting events would have also been observable during the QBA, and thus potentially affected the scoring of certain QBA terms, another second set of video clips were also investigated. This second, separate set involved an additional 63, 1-min video clips that were taken immediately after the QBA clips.

To allow multiple darting events to be recorded in one clip, any darting behaviour must have stopped before the next event could be recorded. The number of salmon involved in each darting event was first recorded and categorised by the proportion to the total number of salmon in the tank (Table 2). Weighted scores were then assigned to each of these categories, relative to their proportions (Table 2). A final score was then calculated for each clip, based on the sum of weighted scores from all darting events recorded. Video playback speed was altered to ensure the number of salmon involved were counted correctly. Where the number of salmon darting was too high to allow for counting, the event was then categorised as involving more than 15% of the fish in the tank.

**Table 2:** Categories of darting events by the proportion of salmon from the tank involved, as well as their corresponding weighted scores.

Proportion of salmon in tank involved in each darting event	Weighted score
Less than 4%	1
Less than 8%	2
Less than 15%	3
More than 15%	4

### 2.5. Statistical analyses

#### 2.5.1. Data handling of QBA scores

For each QBA score, the distance of each observers’ marks from the zero point of the scales was automatically measured and recorded by SurveyMonkey. The complete dataset of these raw QBA scores were then imported from SurveyMonkey into Microsoft Excel (Version 2,301). Data was organised into a matrix, with QBA terms listed horizontally in the first row and video clip numbers and labels in the first few columns. Unless otherwise stated, all statistical analyses were run in R Studio (version 4.2.2). The threshold of significance for any statistical test was *p* < 0.05. For the Linear Mixed Effects Model (LMEM) analyses conducted later in the study, the package “nlme” was applied.

#### 2.5.2. Principal Component Analysis

A Principal Component Analysis (PCA) was carried out, using a correlation matrix on the entire dataset of QBA scores to reduce the dimensionality of the QBA terms. PCA allows for the 16 terms scored within each video clip to be summarised by a numerical value for each Principal Component (i.e., the PC “score”). No post-processing step of ‘rotation’ was carried out, as the only goal of the PCA was to reduce the dimensionality of terms.

The highest positively and negatively loaded terms for each component were identified which, together, represented the larger pattern of expressive characteristics illustrated within each PC. To determine whether PCs were eligible for further analysis, a combination of criteria was used. Following the “Kaiser criterion,” which states that the number of factors to retain should correspond to the number of eigenvalues greater than one, only PCs with eigenvalues >1 were considered (57). Within each component, there also had to be good inter-observer reliability in the PC scores (Section 2.5.3). There also needed to be a coherent biological interpretation of the terms that had the highest positive and negative loadings within each component. For example, a higher score for PC1 suggested that salmon were more unsettled/stressed, whereas a lower score suggested that salmon were more relaxed/content.

For the complete set of PC scores obtained, Q-Q plots, histogram symmetry, skewness and kurtosis values, sphericity, and Leven’s test were inspected to ensure all assumptions required for carrying out further parametric tests were met (including normality of data). The scree plot and proportion of variance for each PC were also used as additional guidance for determining the inclusion of PCs in further analysis.

#### 2.5.3. Inter/intra-observer reliability

Kendall’s coefficient of concordance (W) was used to calculate the level of agreement between the 5 participants’ PC scores in the combined data set, for each of the PCs. Any value of W less than 0.4 was considered to reflect unacceptable inter-observer variability. This analysis was carried out using IBM SPSS Statistics 28 (58). The degree to which observers showed agreement between their scores of the duplicated video clips was, given normal distribution of the scores, determined using Pearson’s correlation, performed on each of the relevant PC scores.

#### 2.5.4. Comparing pre vs. post disturbances

QBA scores of the salmon before and after the stressful challenge were analysed by applying separate Linear Mixed Effects Models (LMEM) to each of the relevant PCs (PC1, PC2, PC3 and PC4). For each LMEM, the PC score was the dependent variable, ‘Pre vs. post disturbance’ and ‘Observer’ were fixed factors, and tank number was a random factor. Before the LMEMs were applied, ANCOVAs were first carried out (with day number as a covariate) to ensure that there were no significant time trends within each subset of days 1–3 and 5–7. Since no additional time trends were present within these subset of days, day number was also included in the LMEMs as a random factor.

Although Kendall’s coefficient determines whether there is good agreement between observers for PC1, PC2, and PC4, the actual “treatment” effect of observers still needed to be accounted for, hence the inclusion of ‘Observer’ as a fixed factor.

#### 2.5.5. Comparing feed intake and darting events with QBA scores

For each tank every day, feed intake and two separate sets of darting scores were recorded (2 x separate sets of 63 video clips). Similar LMEMs were applied, with tank and day number as random factors, to first determine whether ‘Pre vs. post disturbance’ had a significant impact on each of these additional measures. Spearman correlation tests were then carried out to compare feed intake and the two separate sets of darting scores against the corresponding mean PC scores of the 63 clips used in the QBA. Mean PC scores were derived by averaging the PC scores from the 5 observers.

## 3. Results

### 3.1. Qualitative Behavioural Analysis

#### 3.1.1. Principal Component Analysis

PC1, PC2, PC3, and PC4 had eigen values >1. PC1 explained the greatest percentage of variation at 37%, with the first four components collectively explaining 74.5% of the variation in the data (Table 3).

**Table 3:** Eigen analysis of PC1, PC2, PC3, and PC4.

Value	PC1	PC2	PC3	PC4
Eigen value	5.88	2.82	1.95	1.27
% of variation explained	36.7%	17.7%	12.2%	7.9%
Cumulative %	36.7%	54.4%	66.6%	74.5%

**Figure 3:**
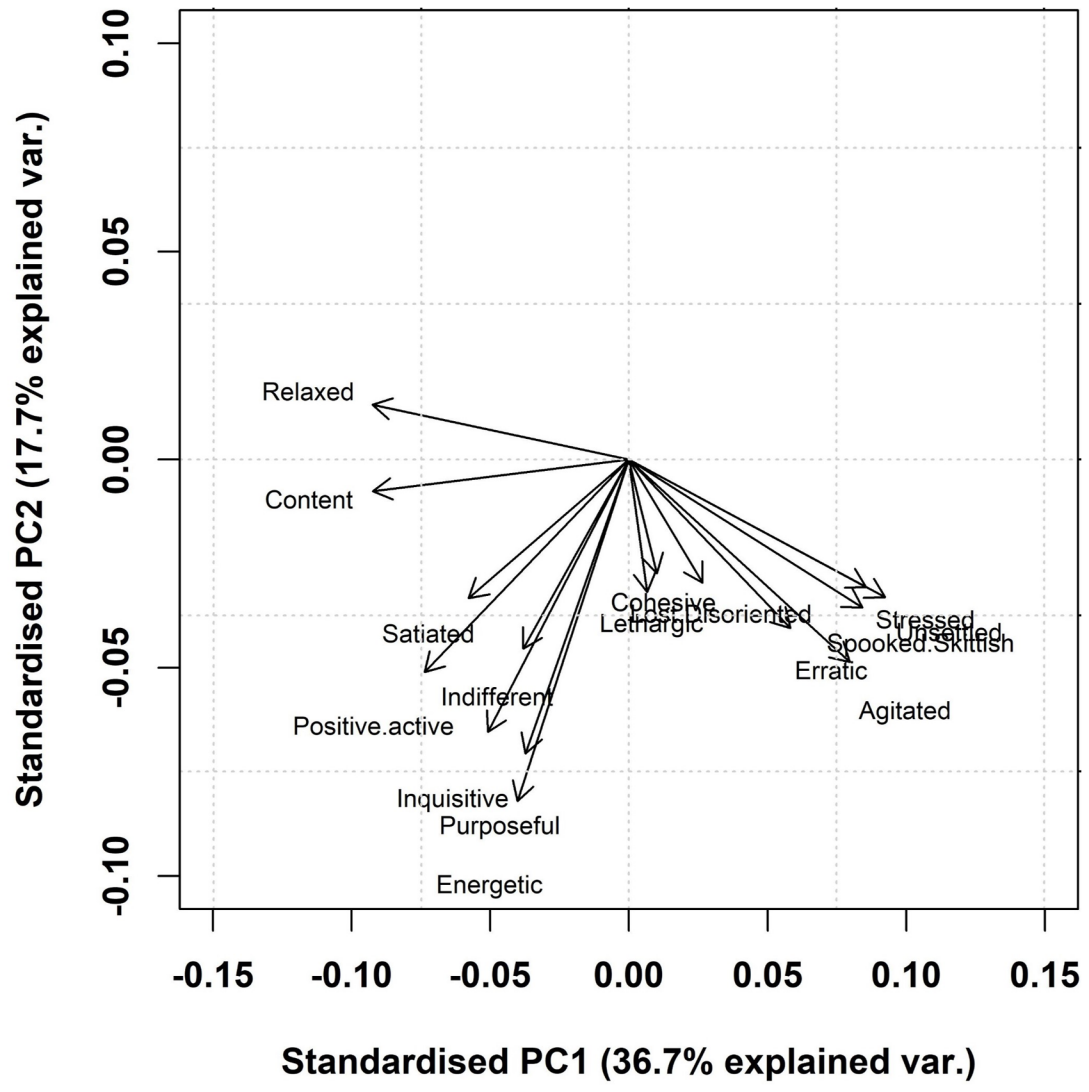
Loading plot of all 16 QBA terms used in this study for PC1 and PC2. Axes represent standardised eigen vectors for which the QBA terms load onto the two main Principal Components of the analysis. A more negative value for PC1 indicates an overall higher score for relaxed, content, and positive active.

As outlined in Table 4, PC1 ranged from relaxed/content/positive active to unsettled/stressed/spooked/skittish/agitated. For PC2, the only positively loading term was relaxed, with the main negatively loading terms being energetic/purposeful/inquisitive. Figure 3 illustrates the relationship that the QBA terms have with both PC1 and PC2. For example, a more negative PC1 score indicates salmon that were more relaxed, content, and positive active.

**Table 4:** QBA term loading values for each principal component.

Term	PC1	PC2	PC3	PC4
Relaxed	**−0.359**	**0.074**	−0.003	0.073
Agitated	**0.309**	−0.272	−0.090	−0.109
Inquisitive	−0.197	**−0.366**	−0.151	0.185
Unsettled	**0.358**	−0.185	−0.073	−0.049
Cohesive	0.039	−0.153	0.297	**−0.491**
Spooked/skittish	**0.327**	−0.199	−0.024	−0.112
Positive active	**−0.286**	−0.286	**−0.168**	0.110
Indifferent	−0.148	−0.254	**0.477**	−0.067
Purposeful	−0.145	**−0.395**	**−0.284**	−0.104
Erratic	0.226	−0.226	−0.038	**0.431**
Energetic	−0.156	**−0.459**	**−0.202**	−0.029
Lost/disoriented	0.103	−0.165	**0.356**	**0.585**
Satiated	−0.224	−0.186	0.232	**−0.290**
Lethargic	0.025	−0.178	**0.560**	0.066
Stressed	**0.332**	−0.171	−0.056	−0.211
Content	**−0.358**	−0.042	0.009	−0.050

The highest negatively and positively loaded terms for each PC are in bold.

PC1, PC2, and PC4 demonstrated acceptable inter-observer reliability for their PC scores (PC1: *W* = 0.63, *X^2^ =* 207.57, *p* < 0.001; PC2: *W* = 0.46, *X^2^ =* 152.19, *p* < 0.001; PC4: *W* = 0.56, *X^2^ =* 184.94, *p* < 0.001). All four PCs showed acceptable intra-observer reliability between PC scores of video clips that were duplicated (PC1: *r* = 0.716, *p* < 0.001; PC2: *r* = 0.755, *p* < 0.001; PC3: *r* = 0.552, *p* < 0.05; PC4: *r* = 0.581, *p* < 0.01). PC3 had a *W* value below 0.4, which was considered unacceptable and therefore not included in further analysis. PC1, PC2, and PC4 were retained for further analysis.

#### 3.1.2. Effect of the stressful challenge (intrusive sampling) on PC scores

**Figure 4:**
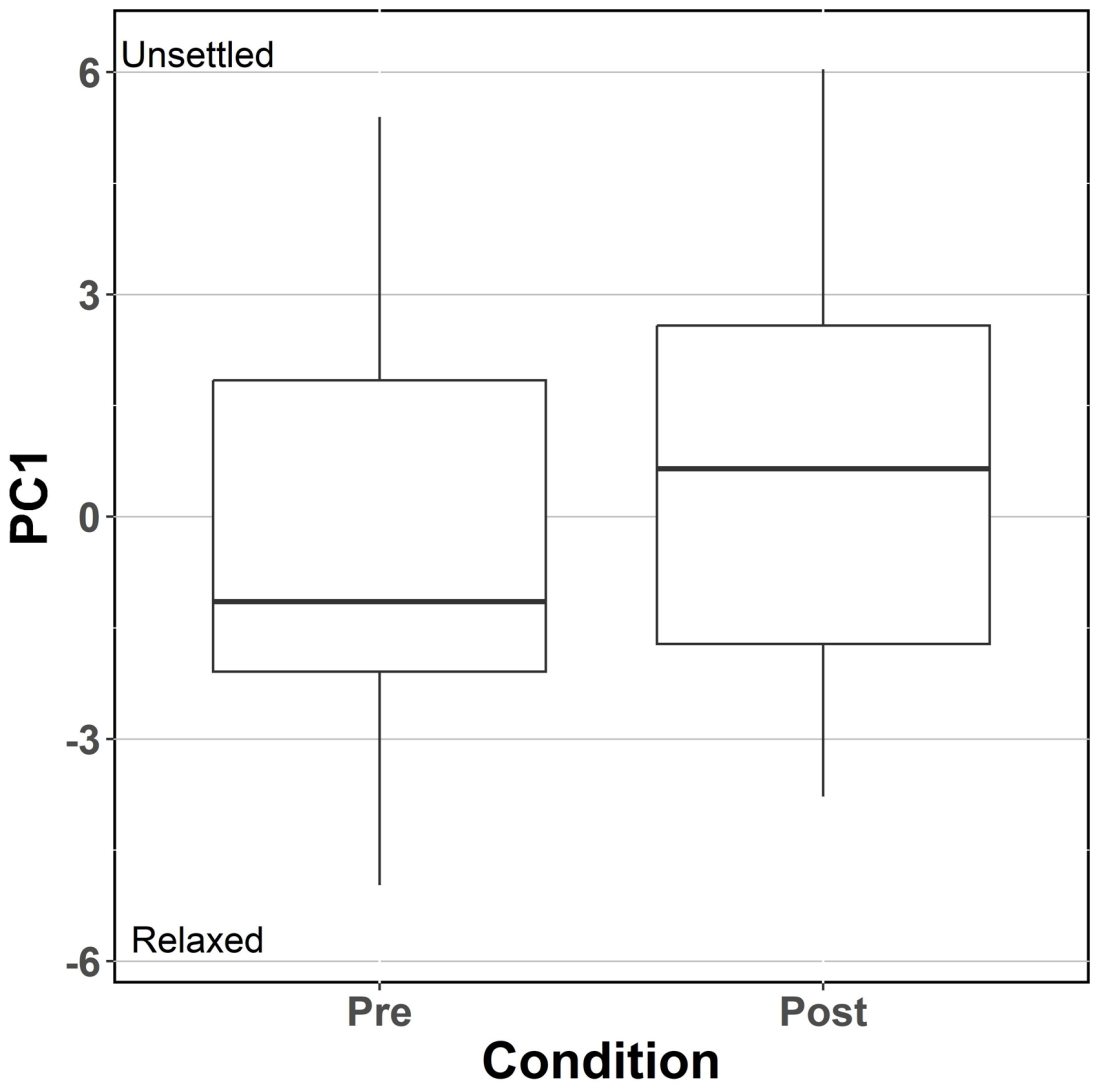
Box plot to compare differences in spread of PC1 scores before and after feed withdrawal and sampling events.

**Figure 5:**
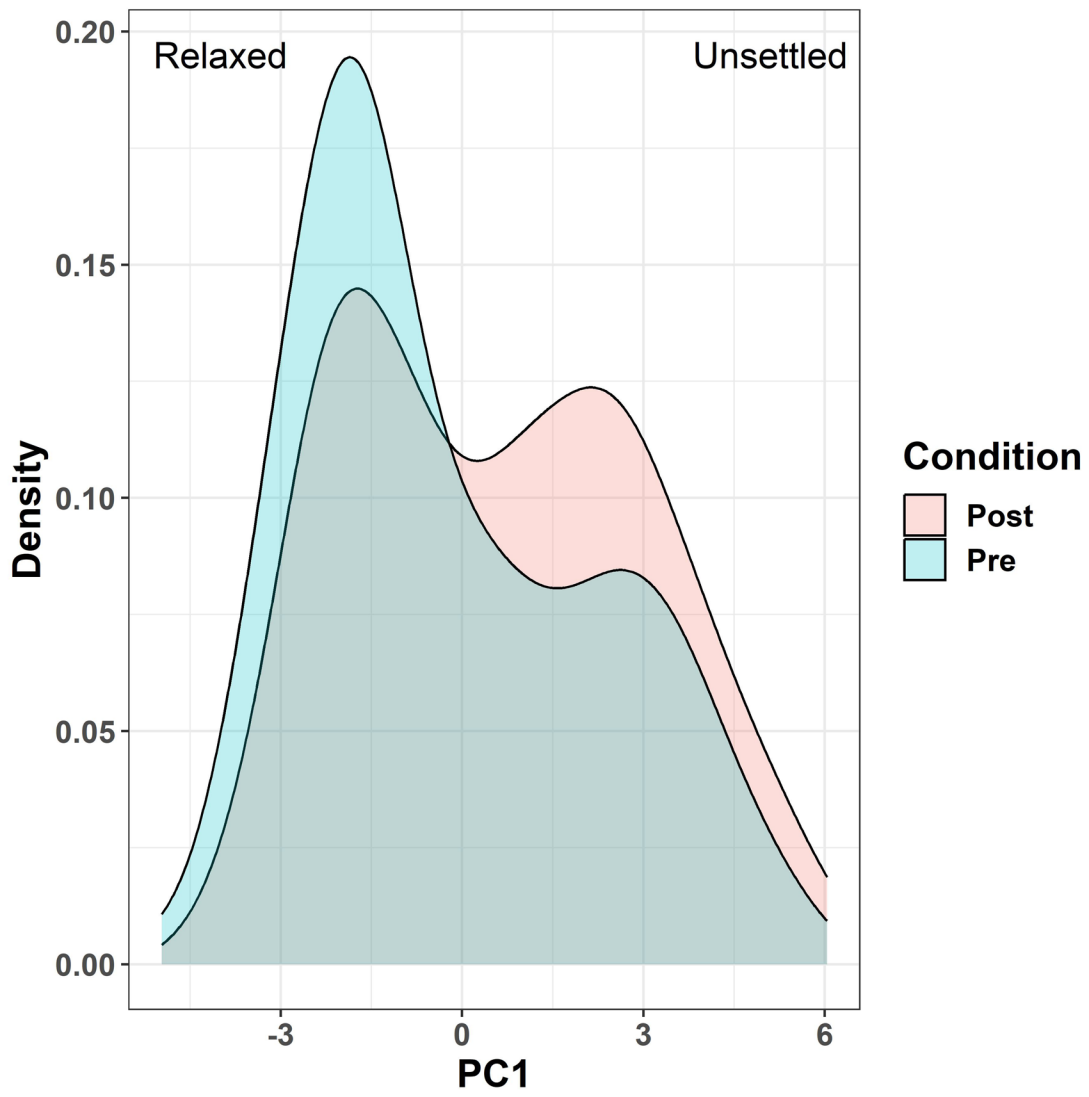
Layered density plot comparing different probabilities of various PC1 scores occurring, depending on whether they were taken pre vs. post disturbance.

There was a significant difference between PC1 scores when comparing days before and after the stressful challenge (*p* = 0.03, Figure 4). PC1 scores (averaged between the 5 observers for each video clip) ranged from −4.97 to 6.04. The mean difference between PC1 scores for pre vs. post-disturbance days was +0.82 (Pre = −0.239, Post = 0.584). Overall, all five observers scored PC1 higher for post-disturbance days. 7 out of 9 tanks received a higher average PC1 score for post-disturbance days. Figure 5 illustrates the comparative likelihood of a PC1 score being higher or lower for video clips that were recorded either before or after the sampling event. No significant differences were found for PC2 and PC4 scores (*p* > 0.05). For PC1, PC2, and PC4, there was a significant effect for observers as a fixed effect (*p* < 0.001).

### 3.2. Feed intake, darting behaviours, and their association with QBA

A significant difference was found in the feed intake of salmon from tanks before and after the stressful challenge (*p* = 0.02). Mean daily feed intakes were 166 g for pre-disturbance (SEM = 4.72) and 79 g for post-disturbance (SEM = 7.5), resulting in an average 87 g reduction in daily feed intake post-disturbance. However, there was no significant association found between mean PC1 scores and feed intake (*r* = −0.19, *p* > 0.05).

**Figure 6:**
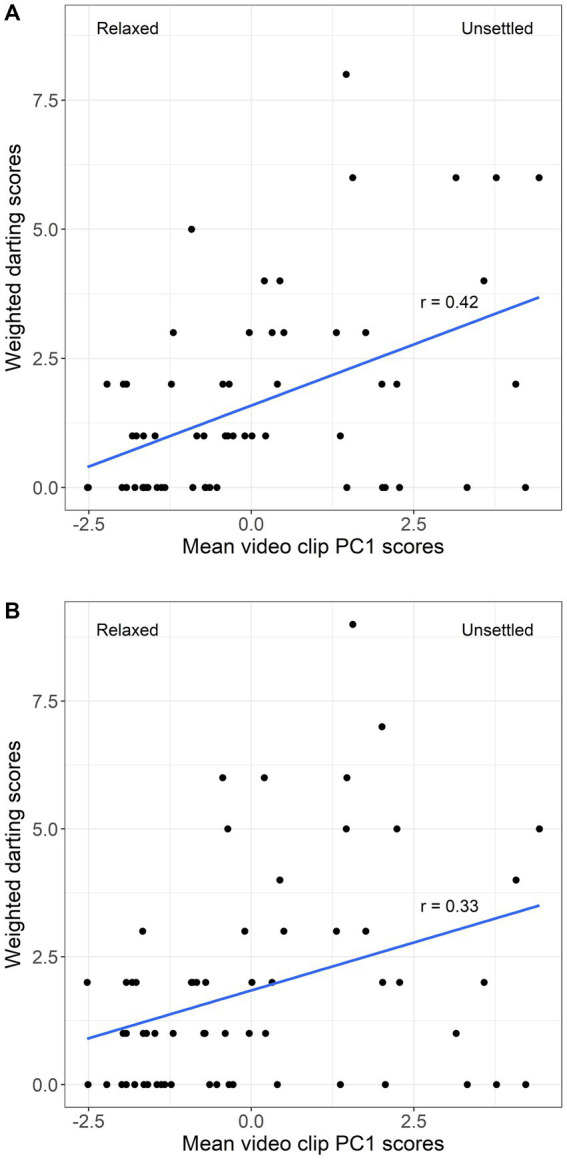
Scatterplot of mean PC1 scores (Relaxed – Unsettled) for video clips vs. **(A)** weighted darting scores calculated from the same clips used for the QBA, and **(B)** weighted darting scores calculated from video clips taken 1-min after QBA clips. Line of best fit and r values from spearman correlation tests included.

No significant difference was found between darting scores before and after the stressful challenge, in either set of 63 video clips used (same clips as QBA: *p* > 0.05; 1-min post QBA clips: *p* > 0.05). However, PC1 scores showed a moderate positive correlation with the darting scores taken from either set of video clips (same clips as QBA: *r* = 0.42, *p* < 0.001; 1-min post QBA clips: *r* = 0.33, *p* < 0.01, see Figure 6).

## 4. Discussion

Integrating indicators of the emotional state of animals within welfare assessments has previously proven to be problematic for many reasons. This study’s aim was to determine QBA’s ability to detect the effects of a stressful event on Atlantic salmon. We applied QBA to quantify and evaluate the expressive characteristics of Atlantic salmon before and after exposure to a putatively stressful challenge. While feed withdrawal was required before sampling could be carried out, the sampling event was the focal point as the experimental treatment of this study. The process of capturing, anaesthetising, and handling salmon out of water for sampling has been described as intrusive, stressful, and detrimental for welfare (59–61). Previous studies that have assessed how salmon recover from stressful events (e.g., handling/anaesthesia/invasive sampling) often monitored the recovery over a 24-72 h period (59, 62, 63). Thus, a 3-day period for both the baseline and ‘recovery’ stage was considered to be sufficient for the purpose of this study.

There was acceptable agreement between the five observers in this study, who were blind to the treatment and had varied experience in monitoring fish behaviour/welfare. There was one main dimension of QBA that proved effective in capturing changes in the emotional state of the salmon within this study; relaxed/content/positive active – unsettled/stressed/spooked/skittish/agitated (PC1). PC1 explained the largest proportion of variation in expressive characteristics of the salmon (36.7%). There were significant differences between PC1 scores before and after the stressful challenge (sampling), with salmon being scored as more unsettled/stressed/spooked/skittish/agitated after sampling. This reflected a shift from low energy, positive valence to high energy, negative valence after sampling, a contrast that was consistently recorded by all observers and in the majority of tanks. In addition, the single recording that was perceived the most “positively” (i.e., the most relaxed/content/positive active) was taken before any potential impacts from sampling had occurred. These results are in agreement with numerous papers that have previously used QBA to assess the emotional state of terrestrial farmed animals (e.g., for cattle, horses, pigs, and hens), with PC1 typically being characterised by terms such as relaxed and content vs. agitated (64–68). Furthermore, these past studies have used similar descriptors to describe the other main terms used in PC1 for this study; unsettled (uneasy), stressed (nervous), spooked/skittish (scared/fearful/nervous), and stressed (tense).

With lights being switched on at precisely 10:30 am every morning, this was considered a routine event that could be methodically recorded and expected to help stimulate activity in the fish. This would potentially maximise what expressive characteristics could be captured without causing additional stress to the salmon. The initial 30 s were cut out to exclude the salmon’s startle responses to the lights, which may have otherwise drowned out any potential differences reflected by the QBA scores.

The LMEM determined that there was significant variation between observers in the mean scores they attributed to the 63 video clips on each PC. This suggests that observers may have been interpreting and using the ranges within the VASs differently, while still agreeing on the direction in which the scores should change from one video to another. Such an occurrence is not uncommon when multiple individuals use the same continuous scales (69). In most QBA studies, the directionality of scores, as indicated by Kendall’s W, is taken as the most important indicator for inter-observer agreement (17, 44, 70). However, crucial to the aims of this study, the observer effect was accounted for by the LMEM when analysing the treatment effect, and thus a significant difference between PC1 scores was found before and after the stressful challenge.

Previous studies have suggested that significant associations between QBA and other welfare measures help support the validity of QBA as a welfare assessment tool (17, 36, 44, 68). However, as noted by (37), the purpose of QBA is to examine subtle expressive aspects of an animal’s demeanour in ways that would be otherwise difficult to quantify for other measures of behaviour. It is important to be reminded of the multi-faceted nature of welfare (16, 71, 72), and that QBA should be regarded as a complementary addition to an integrated approach involving various welfare indicators. QBA is thus used with the intention of gaining unique insights into an animal’s emotional state in a way that is complementary to other indicators, allowing for a more comprehensive evaluation of animal welfare (17, 37). Welfare assessments should also aim to minimise redundancies and include measures that are, at least to some degree, independent from each other (73). Feed intake, on average, more than halved following the stressful challenge. Similar reductions in feed intake have been reported in a number of studies exposing fish to stressful challenges (28, 53, 74). As there were significant differences in both PC1 scores and feed intake before and after the stressful challenge, and yet they were not correlated with each other, these results should further support the notion of QBA being a unique welfare assessment tool. In somewhat of a contrast to this, darting scores showed a moderately positive correlation to PC1 scores. Put simply, as the salmon were observed to be more unsettled, stressed, spooked/skittish, and agitated, there was a corresponding increase in the frequency and/or intensity of darting events. However, the darting scores alone showed no treatment effect from the stressful challenge. While these two measures were not entirely independent from one another, QBA was capable of capturing a significant treatment effect when the darting scores could not. This finding highlights the sensitivity of QBA, indicating that the PC1 scores were more capable of capturing the effects of the stressful challenge on the salmon’s welfare than the darting scores.

PC2 and PC4 showed acceptable inter-observer reliability, explaining proportions of variation that were comparable to other studies applying QBA to terrestrial animals (36, 44, 75, 76). For PC2, the only positively loading QBA term was relaxed, with the main negatively loading terms being energetic, purposeful, and inquisitive. This meant that PC2 mainly reflected the salmon’s degree of relaxation against ‘high energy’; lower PC2 scores reflected more lively, energetic salmon. PC4 was characterised by terms that reflected a shift in how “harmonious” or “consistent” the behaviour of the salmon was as a collective (i.e., cohesive vs. lost/disoriented). PC3 explained one third of the proportion of variation explained by PC1, with poor inter-observer reliability. The terms most heavily loaded for this dimension (indifferent and purposeful) may help partially explain this inconsistency between observers. Such terms could have been more difficult to perceive and assess in salmon, in comparison to the terms used within PC1.

There was no statistically significant difference between the pre- and post- sampling event stages in PC2 or PC4. Sampling was specifically chosen as a presumably intrusive, stressful event, with the intentions of then assessing QBA’s ability to detect the putative impacts of such an event on the salmons’ emotional state. Considering the terms used to characterise PC2 and PC4, these dimensions may not be so relevant to addressing the effects of stress, but may be very relevant to assess fish welfare in other contexts and treatments. For example, the potential benefits of environmental enrichment or the impacts of transportation/transfer to new enclosures. Considering that the most relevant dimension in the context of this study (PC1) reflects a combined shift in both valence (positive – negative) and energy (low – high), this dimension could be of significant use for on-farm welfare assessments of Atlantic salmon. Additional research is needed to further explore and validate the relevance of other dimensions found in this study (i.e., PC2 and PC4), under different experimental treatments, to expand the potential applications of QBA for salmon welfare assessments.

Integrating QBA into future welfare assessments (for research or farming) will first require appropriate training in the observing, scoring, and understanding of terms involved (70, 77). While this may require a significant initial investment towards developing the observers’ assessment capabilities, doing so will help ensure acceptable inter-observer reliability and, over the long term, help with the integration of a unique and efficient welfare assessment tool (17). Welfare assessments that include QBA have the advantage of evaluating emotional states of the animals, and the consequent monitoring of positively valenced terms (e.g., content, relaxed, inquisitive, cohesive, purposeful, energetic etc.) also allows for the consideration of positive aspects of fish welfare.

The various ways in which sampling can cause stress and impair fish welfare demonstrates another advantage with implementing QBA; as a non-intrusive method of welfare assessment. QBA avoids any negative impacts from its measurement, an issue that is inherent in many animal-based measures. A large proportion of animal-based measures of welfare are also retrospective, only identifying problems long after they have occurred (72). Analyses of behavioural expression could help minimise this delay, perhaps even to the point of providing early warning signs for pre-clinical health issues (13). QBA has for example been used successfully to detect early clinical signs of mastitis in dairy cows (78). Through virtue of being able to assess behavioural expressions through video monitoring, QBA is also capable of being carried out remotely. Considering the remote locations in which these salmon are often kept (79), as well as issues surrounding monitoring when site access is limited, this feature provides a significant advantage. The need for such welfare monitoring tools was highlighted to the Scottish salmon farming sector when farm staff were restricted from accessing their sites during the 2020 COVID-19 pandemic, and in-person audits for welfare certification schemes had to be replaced with virtual assessments for 2 months (80, 81). During a recent industry survey carried out within the salmon farming sector, various professionals employed in the production process ranked the development of remote, non-intrusive welfare indicators as one of the highest research priorities for farmed salmon welfare (32). The effective implementation of QBA on-site would help meet this demand.

## 5. Conclusion

This is the first study to demonstrate QBA’s ability to capture changes in the expressive characteristics of Atlantic salmon following exposure to putatively stressful events. Five observers from various professional backgrounds achieved acceptable inter- and intra- observer reliability in 3 dimensions of QBA scores. PC1 showed a significant treatment effect, with salmon becoming more unsettled, stressed, spooked/skittish, and agitated after the stressful challenge. Both PC1 scores and feed intake recorded a significant difference before and after the stressful challenge, but were not correlated to each other. PC1 scores showed a moderate positive correlation with darting scores, however the darting scores did not show a significant treatment effect, indicating the QBA scores to be more sensitive to the stressful challenge. These results support QBA’s ability to provide unique insights that are relevant to the evaluation of farmed salmon welfare. Future experiments should explore the other dimensions found within QBA (e.g., PC2 and PC4) under different treatment conditions, and across other species of fish, to further investigate QBA’s applicability within aquaculture. The results from this study demonstrate that QBA is a promising welfare indicator that, with further research, could act as a time-efficient and complimentary tool for on-farm welfare assessments.

## Data availability statement

The raw data supporting the conclusions of this article will be made available by the authors, without undue reservation.

## Author contributions

TW: Conceptualization, Data curation, Formal analysis, Investigation, Methodology, Visualization, Writing – original draft, Writing – review & editing. SR: Conceptualization, Supervision, Validation, Visualization, Writing – review & editing. MB: Methodology, Validation, Writing – review & editing, Data curation, Resources. MH: Conceptualization, Funding acquisition, Methodology, Supervision, Validation, Visualization, Writing – review & editing. SJ: Validation, Writing – review & editing, Conceptualization, Funding acquisition, Methodology, Supervision, Visualization. AD: Validation, Writing – review & editing. FW: Conceptualization, Validation, Writing – review & editing, Methodology. JT: Conceptualization, Funding acquisition, Supervision, Validation, Visualization, Writing – review & editing.

## Funding

The author(s) declare that no financial support was received for the research, authorship, and/or publication of this article.

## Conflict of interest

AD was employed by Aquascot Ltd.

The remaining authors declare that the research was conducted in the absence of any commercial or financial relationships that could be construed as a potential conflict of interest.

## Publisher’s note

All claims expressed in this article are solely those of the authors and do not necessarily represent those of their affiliated organizations, or those of the publisher, the editors and the reviewers. Any product that may be evaluated in this article, or claim that may be made by its manufacturer, is not guaranteed or endorsed by the publisher.
